# Hepatitis C in HIV-infected individuals: cure and control, right now

**DOI:** 10.1186/1758-2652-14-22

**Published:** 2011-05-08

**Authors:** David L Thomas, Dennis Leoutsakas, Tomas Zabransky, M Suresh Kumar

**Affiliations:** 1 Johns Hopkins School of Medicine, Baltimore, USA; 2 Salisbury University, Salisbury, Maryland, USA; 3 Center for Addictology, Department of Psychiatry, First Faculty of Medicine, Charles University in Prague and General University Hospital, Prague; 4 Gaitonde Centre for AIDS Research and Education, Chennai, India

## Abstract

For persons living with HIV, hepatitis C is a major public health problem that must be controlled and could be eliminated. The challenge arises because the hepatitis C virus (HCV) is prevalent among HIV-infected persons in most parts of the world, because HIV worsens all HCV outcomes, and because HCV may add additional individual economic and psychosocial complications to HIV disease. Despite the major benefits of antiretroviral therapy on HIV outcomes, antiretroviral therapy is not sufficient to halt the complications of HCV. Nonetheless, HCV can be controlled at all stages, including prevention of infection and cure. Thus, HCV is an eradicable disease. There are significant inequalities worldwide in HCV control that could markedly constrain the impact of these measures.

## Review

### Public health importance

#### HCV coinfection is common

**Figure 1:**
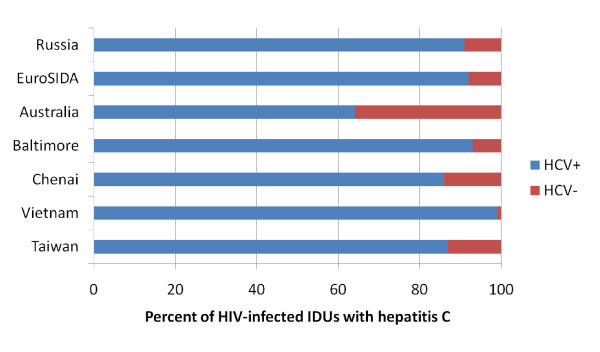
**HCV co-infection is common among HIV-infected injecting drug users**.

Many persons living with HIV (PLHIV) are coinfected with the hepatitis C virus (HCV). In Europe, the United States and Australia, approximately one-quarter of PLHIV are coinfected with HCV. But these averages mask marked differences in HCV prevalence based on the route through which HIV was acquired. By far, HCV coinfection rates are highest among persons who have acquired HIV from injection drug use (Figure 1).

Rockstroh and coworkers reported in the large EuroSIDA cohort that 1519 (92%) of HIV-infected persons with a history of injecting drug use were also HCV exposed [1]. In one study from Russia, 91% of HIV-infected injecting drug users (IDUs) had HCV antibodies [2]. Similarly high HIV/HCV coinfection prevalence rates have been reported from the USA and Australia [3,4].

In Asia also, nearly all HIV-infected IDUs also has HCV. For example, in IDUs from Chennai, India, the prevalence of coinfection with anti-HCV was 86% [5]. Among IDUs in northern Vietnam, almost all (98.5%) HIV-infected IDUs were also co-infected with HCV [6]. Likewise, HCV infection has been reported in more than 90% of IDUs in some regions in southern China [7,8]. Given the linkage with illicit drug use, HCV is also a special problem for PLHIV in prisons [9]. In addition to risk-prone illicit drug use, unsafe tattooing contributes to HCV infection in prisons [10].

In contrast to IDUs, HCV coinfection occurs in less than 15% of persons who acquired HIV from sexual intercourse [1,11]. One important caveat is the recent series of HCV outbreaks among HIV-infected men who have sex with men (MSM) [12,13]. For example, Danta and coworkers described 111 acute HCV infections among MSM in England that were associated with high-risk sexual practices, as well as non-injection use of illicit drugs [12]. It is now clear that this is a widespread international trend [14].

#### Basis for HIV/HCV coinfection

There are biologic, sociologic and historic reasons that HCV coinfection is common in HIV-infected IDUs. Studies following a single needlestick exposure show that HCV is approximately a log_10 _more transmissible than HIV (1/100 versus <1/1000) [15]. With transfusion of large blood volumes, such as occurred in haemophilia populations in the early 1980s, these differences in transmissibility were inconsequential. Nearly all who were exposed by transfusion became infected [16].

**Figure 2:**
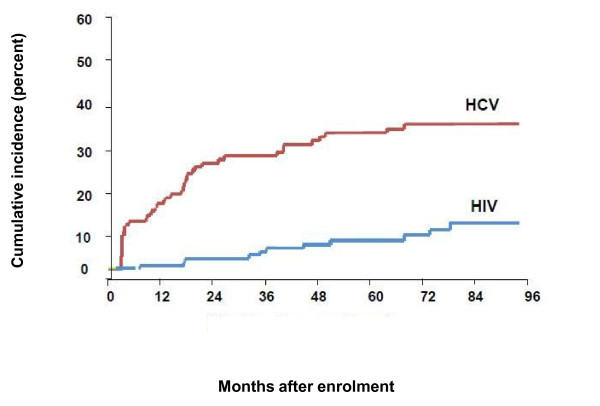
**Incidence of HIV and HCV among Baltimore IDUs**. Adapted from Villano et al [46].

However, transmission of infectious diseases by drug use typically involves inocula that are 4-5 log_10 _smaller. The average HIV plasma viral load is approximately a log_10 _less than HCV [17,18]. Thus, with the very small inocula that are typically involved in injection drug use, it is likely that the number of infectious virions in blood is a major factor in the greater transmission of HCV compared with HIV. It is also possible that each HIV virion is less transmissible or less environmentally stable than HCV. Ultimately, the net percutaneous transmissibility of small blood inocula is lower for HIV than for HCV and a major reason for the greater HCV incidence and prevalence among IDUs (Figure 2).

Less is known about the basis for the sometimes contradictory evidence regarding sexual transmission of HCV. For example, there are higher than expected HCV prevalence rates in populations with high-risk sexual practices, including MSM, but there is very low HCV incidence between discordant couples and in some MSM populations. It is possible that, like HIV, HCV is more transmissible just after acute infection, before formation of neutralizing antibodies and when viral titers are higher.

However, while this feature might explain low/no sexual transmission from persons with chronic hepatitis C and relatively more transmission in settings where multiple exposures to acute infections occur, there is no evidence to support the conjecture. One explanation for the recent rise in transmission among MSM is that antiretroviral therapy has reduced the fear of HIV, resulting in higher risk sexual practices than when HIV was uniformly fatal. Increased use of illicit drugs by injection or non-injection routes may also contribute. In addition, it is also possible that there is simply a greater reservoir of HCV infection among MSM than in prior years, increasing the likelihood of exposure.

Historically, the population reservoir of HCV also antedated HIV. HCV prevalence markedly expanded during the mid1900s and is strongly correlated with stepped-up production of syringes and their worldwide use both for conventional and illicit drugs [19-21]. This trend allowed HCV to spread throughout the world and explains the five- to 20-fold increased HCV prevalence rates in certain regions where unsafe injections were widespread, and among IDUs [21]. There is a tendency of older IDUs to initiate new users by inoculating themselves first. Since older IDUs are often HCV infected, this practice contributes to rapid acquisition of HCV among IDUs [22]. HIV infection has overspread IDU populations more recently than HCV which was distributed decades earlier.

In Baltimore, the HIV prevalence in various risk groups was extremely low in 1980 and rose sharply in the early 1990s [23,24]. Consequently, most HIV/HCV-coinfected IDUs acquired HCV years before HIV, and for every one HIV/HCV-coinfected IDU, there are usually two others infected with HCV but not HIV. In Asia, HIV incidence and prevalence rates first rose in the mid and late 1980s, especially among IDUs. While there are certainly individual instances in which HIV infection occurred first, in most populations in which injection drug use antedated the introduction of HIV, IDUs were already infected with HCV. Since HIV infects activated T cells, the immunologic control of an infectious disease like HCV may differ if it first occurs after a person is HIV infected, and that consideration may explain differences in HCV natural history and seroreversion rates in various studies.

#### HIV infection complicates the course of hepatitis C

**Figure 3:**
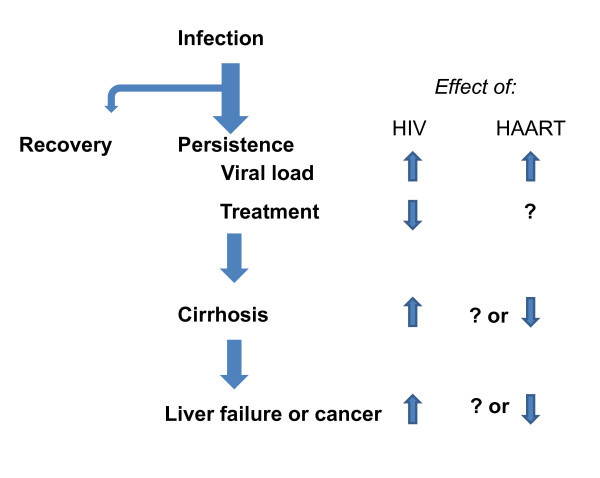
**HIV infection adversely affects all stages of hepatitis C**.

Although the mechanism is really not well understood, HCV infection has a well-characterized course that is adversely affected at every stage by HIV (Figure 3). Although 30% to 40% of persons spontaneously clear HCV infection, the chances of spontaneous resolution are at least two-fold lower in HIV/HCV-coinfected persons [25]. Those who do develop chronic hepatitis C have higher HCV viral loads if they are coinfected with HIV than those with just HCV [26].

Likewise, persons chronically infected with the same HCV genotype are about half as likely to respond to the same dose and duration of peginterferon and ribavirin therapy if they are HIV coinfected compared with those with just HCV [27,28]. HIV/HCV-coinfected persons are also twice as likely to develop cirrhosis over a shorter period of time [29]. Clinical manifestations of liver failure, such as ascites, encephalopathy and esophageal varices, all occur more often in HIV/HCV-coinfected persons [30].

#### Antiretroviral therapy is not sufficient to negate the effects of HIV on the course of hepatitis C

Antiretroviral therapy is probably the most important biomedical breakthrough in the past two decades. Rates of HIV-related opportunistic infection and related mortality have sharply dropped since adoption of antiretroviral therapy in the mid 1990s. Antiretroviral therapy also worked in sub-Saharan Africa and wherever there was a public health system to deliver it. Transmission of HIV to infants is also dramatically reduced in settings where antiretroviral therapy is given. Even the relatively hard-to-tolerate first-generation antiretroviral therapy reduced mortality.

The enormous effectiveness of antiretroviral therapy and the observation that HIV makes liver disease worse raises the question of whether antiretroviral therapy eliminates or at least attenuates the effect. This is an intense area of investigation with too much data on both sides of the question to review here. What is clear is that antiretroviral therapy *is not sufficient to *prevent complications of HCV, antiretroviral therapy does not affect the rate of HCV persistence, and it does not diminish the HCV viral load (in some persons, antiretroviral therapy actually increases the HCV-RNA level by a small amount) [31].

Antiretroviral therapy might improve the response to interferon and ribavirin, but this hypothesis has never been appropriately tested, and, since the majority of published data on peginterferon/ribavirin response in HIV/HCV-coinfected persons are on persons taking fully suppressive antiretroviral therapy, it is clear that "normal" responses are not restored. Likewise, rapid progression of liver fibrosis has been described even in persons taking fully suppressive antiretroviral therapy, making it clear that antiretroviral therapy is not sufficient to control HCV infection in PLHIV.

**Figure 4:**
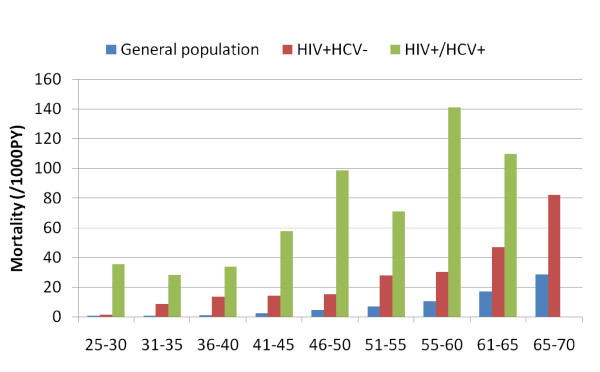
**Markedly lower survival for HIV/HCV-coinfected persons in Denmark: 2000-2005**. Reproduced from Lohse *et al *[32].

While the lifespan of HIV-infected persons taking antiretroviral therapy has been prolonged, the lives of those coinfected with HCV remain much shorter. In one study from Denmark, mortality rates from the age of 25 years were compared in 3990 HIV-infected persons with the general population, and survival curves were fitted [32]. Mortality dropped from a high of 124/1000 person years (PYs) in the pre-antiretroviral therapy period to 38/1000 PYs in 1997-1999 to 25/1000 PYs in 2000-2005. However, the impact was markedly different by HCV coinfection status, with mortality rates in 2000-2005 of 57/1000 PYs among the HCV coinfected compared with 19/1000 among those without known HCV infection (Figure 4).

By removing the HCV-coinfected persons, survival of HIV-infected persons on antiretroviral therapy was expected to approach that of the general population, 38.9 versus 51.1 years. Some of this is caused by other health problems, like overdose or accidents. However, liver disease is now the second leading cause of death among persons with HIV taking antiretroviral therapy [33,34]. Given the systematic underreporting of liver disease, the true impact is likely greater.

#### Beyond statistics

There are consequences of HCV infection that are often not appreciated by physicians and public health officials. Since HCV often comes from injecting drug use, ongoing infection can be an indelible scar of a difficult phase of life. Having overcome dependency and other related challenges and having achieved an undetectable HIV-RNA on antiretroviral therapy, HCV can represent a major residual concern for PLHIV.

There may also be stigma associated with being HCV infected [35]. In some societies, this can be extremely severe. For those who have been undergoing HIV treatment, or have been in search of appropriate HIV drug combinations, the addition of an HCV regimen can be discouraging. Often already having undergone a battle with the HIV medications, coinfected persons may not emotionally or psychologically embrace the addition of highly toxic drugs to already complex treatment plans [36]. Patients have various levels of tolerance, which together with the different side-effect experiences, may produce an array of responses from little perceivable impact on daily functions to major suffering and outright non-compliance. The majority of patients, of course, fall somewhere in between, and none enjoy HCV treatment.

Once diagnosed with HCV, IDUs who historically associate needle use with illicit drug use, now must inject themselves with a compound that often exacerbates depression [27]. For newly recovering addicts, this process, along with the HCV diagnosis, can be detrimental to their efforts to maintain abstinence from mood-altering drugs, and, therefore, they may even refuse to participate, become non-compliant, or return to illicit drug use. For some of the co-infected persons, especially young HIV-positive persons, not responding to treatment constitutes a failure of modern medicine to meet their health needs. This realization may produce all types of psychological responses, like self-blame, severe depression or misplaced rage.

### Control of HCV infection

Proof-of-principle studies have demonstrated that all phases of control are possible, from prevention to treatment. At the same time, most research suggests that HCV control will create huge disparities in outcomes.

#### HCV transmission can be prevented

HCV transmission can clearly be prevented. Transfusion transmission of HCV has virtually been eliminated in any setting where donations are screened for HCV antibodies and RNA [37]. Likewise, nosocomial transmission has been reduced by observance of blood-borne precautions.

#### Prevention of HCV transmission among IDUs can be achieved but is more difficult than HIV

Harm-reduction efforts, such as needle exchange, have been associated with reductions in HIV and HCV incidences in some settings. In Baltimore, HIV incidence rates among IDUs have dropped markedly from ~5 per 100 person years in 1990 to nearly zero since 2000. In the same Baltimore cohort, HCV incidence dropped from 22 to eight per 100 person years over the same time span (Mehta S, personal communication).

Given the greater transmissibility of HCV and the greater reservoir of IDUs already infected (as we have discussed), it is not surprising that even stronger harm-reduction measures will be needed to eliminate new HCV infections. If HCV is a log_10 _more transmissible than HIV, public health interventions may need to be an order of magnitude stronger to control HCV.

#### Control of chronic hepatitis C

HCV infection can also be controlled even after it occurs. The first step in controlling chronic hepatitis C is detecting infection by screening and testing. Nearly all guidelines indicate that HIV-infected persons and all IDUs be tested for HCV [38-40]. However, the effectiveness of such guidelines and the associated practices varies markedly.

In the USA, rates of testing and awareness are lower. Some have estimated that 70% of all those with chronic hepatitis C in the USA are unaware of their infection. Data from The TREAT Asia (Therapeutic Research, Education and AIDS Training in Asia) HIV Observational Database, a collaborative observational cohort study involving 15 participating sites in 12 cities in the Asia and Pacific region, indicate that hepatitis testing data are available in nearly half of the TAHOD patients, with prevalence of HBV and HCV coinfection each at approximately 10% [41]. Lack of access to HCV testing and counseling is a major problem throughout eastern and central Europe and in Asia. The cost of the test is a major barrier.

Stigmatization of illicit drug use and HCV infection itself are obstacles to screening programmes. However, these issues have been addressed for HIV and can be overcome by a variety of measures that are likely to differ in various cultures.

#### Opting for HCV testing

The prevalence of HCV infection is sufficiently high to justify testing of any IDU and the stigma of acknowledging illicit drug use is sufficiently high that routing testing may be preferable to screening for specific HCV risk factors (and then testing those with risk). HCV testing should include counseling and, ideally, opportunities for disease management, which sometimes will involve treatment.

Given the small proportions of HCV-infected persons who are currently aware of their infections, greater effort also should be given to development and implementation of rapid HCV tests. For example, tests that can be used at any venue and provide results that can be interpreted at the same visit are highly desirable, as with HIV [42]. Counseling to reduce transmission to others and to reduce the harm of alcohol use is essential. Vaccination should be provided to susceptible persons to prevent hepatitis A virus and hepatitis B virus infections [39,40].

#### Treatment is the most potent form of controlling chronic hepatitis C

HCV infection can be cured. Even in persons with HIV, suppression of replication during treatment and for six months after treatment (called a sustained virologic response, or SVR) is considered a cure because more than 98% will remain free of HCV viremia off medication for five or more years [43]. In addition, end-stage liver disease, liver cancer and other complications of HCV infection are reduced [44].

The global standard of care is peginterferon alfa and ribavirin, a combination that is expensive, toxic and effective approximately 50% of the time. Thus, guidelines currently recommend that treatment be used only for persons most likely to benefit (high disease stage, low risk of adverse events). However, treatment improvements are coming rapidly. "Highly active" HCV therapies are being developed, and approval of new compounds (often called direct acting agents, or DAAs) is expected in 2011. It is already clear that DAAs improve cure rates and will probably also reduce treatment durations [45]. In addition, clinical trials of interferon-free treatments are already in phase 2. Thus, it is reasonable to anticipate a day when 90% of HCV-infected persons could be cured by 24 weeks or less of treatment.

#### Even elimination of HCV is possible

There are many reasons why HCV could (and should) be eliminated. Humans are the only reservoir of HCV infection. Unlike HIV or HBV, there is no latent reservoir of HCV. When a patient is cured, they are no longer infectious to others. If environmental reservoir is reduced enough, infection will not be sustained. It is simply a matter of better drugs, which are anticipated, and then the allocation of resources to treat a sufficient number to break the transmission chain.

### HCV outcome disparities

It is likely that existing inequities in HCV outcomes will expand markedly as therapies improve. Discrepancies already exist in the prevention and treatment of HCV infections worldwide. HCV transmission by transfusion has been eliminated from the USA and Europe, but continues in areas in the world in which donations are not screened effectively. HCV infection can be diagnosed, but fewer than 10% of the 170 million persons with HCV worldwide are aware of their infections. Even fewer have the opportunity for treatment.

In an interview with the United Nations Office on Drugs and Crime, Loon Gangte, President of the Dehli Network of Positive People, observed that the price of peginterferon and ribavirin exceeds the lifetime income of most Indians http://www.unodc.org/india/interview-with-mr.-loon-gangte.html.

## Conclusions

In this way, HCV is much like HIV in the mid 1990s. HCV is about to enter an exciting era of more potent treatment that will reduce the mortality and morbidity associated with the infection. Treatments will continue to improve as newer generations of medications are developed. Like HIV, the benefits of treatment will be narrow unless there is a global response, such as what has occurred to bring antiretroviral therapy to millions of additional persons worldwide.

**Figure 5:**
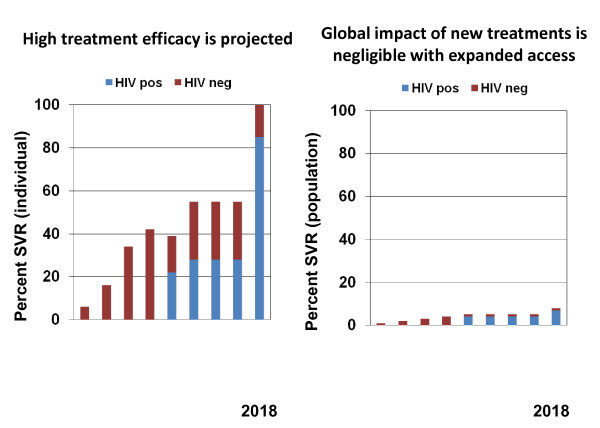
**The disparity between potential HCV treatment efficacy and projected HCV treatment effectiveness**. Adapted from Thomas DL [47].

Will there be a Global Fund or PEPFAR for HCV? Even if all those who take HCV treatment are cured, if fewer than 10% receive care, the global impact of treatment remains minimal (Figure 5). The effectiveness of efficacious therapy will be low.

On 21 May 2010, the World Health Assembly passed a resolution that called for the World Health Organization to observe World Hepatitis Day on July 28 and to develop a comprehensive approach to control of chronic hepatitis. This is an exciting first step. However, a tremendous amount of work still needs to be done to bring new HCV treatments to those who need them and, perhaps some day, to eradicate another global infectious disease.

## Competing interests

DLT has served as a scientific advisor to Merck and has received from Gilead and Merck antiretroviral therapy to support research studies. The other authors have no competing interests to declare.

## Authors' contributions

All authors contributed to the concept of the work and reviewed the paper. DLT chiefly wrote the paper. MSK and DT performed the research that contributed to the figures cited. All authors have read and approved the final manuscript.
